# The application of explainable machine learning for the identification of MASLD in non-obese populations

**DOI:** 10.3389/fpubh.2026.1851486

**Published:** 2026-09-28

**Authors:** Huiyan Yu, Xiuli Han, Nayi Zhang, Kepeng Xu, Jie Liu, Yao Zhong, Xiaoyu Wang, Yuxia Qi

**Affiliations:** 1 Department of Critical Care Medicine, Qingdao Central Hospital, University of Health and Rehabilitation Sciences (Qingdao Central Hospital), Qingdao, China; 2 General Internal Medicine, Sixth People's Hospital of Qingdao, Qingdao Public Health Clinical Center, Qingdao, China

**Keywords:** Gradient Boosting Machine, Linear Discriminant Analysis, machine learning, metabolic dysfunction-associated steatotic liver disease, non-obese, SHAP

## Abstract

**Background:**

The diagnosis of metabolic dysfunction-associated steatotic liver disease (MASLD) in non-obese individuals presents a significant challenge, and its early identification is of considerable public health importance. This study aimed to develop and validate interpretable machine learning models using routine clinical parameters to identify MASLD in this specific population.

**Methods:**

Data from 768 non-obese subjects were analyzed. The dataset was randomly split into training (*n* = 615, 80%) and testing (*n* = 153, 20%) sets using stratified sampling. LASSO regression was performed within the training set for feature selection. Eight machine learning models were constructed in the training set: Linear Discriminant Analysis (LDA), Gradient Boosting Machine (GBM), Neural Network (NN), Logistic Regression (LR), Support Vector Machine (SVM), Naïve Bayes (NB), Random Forest (RF), and Decision Tree (DT). Model performance was evaluated using AUC, calibration analysis and decision curve analysis (DCA). SHAP analysis was applied to interpret the optimal models.

**Results:**

LASSO regression identified eight factors: apolipoprotein B, triglycerides, fasting insulin, fasting plasma glucose, waist circumference, age, alkaline phosphatase, and systolic blood pressure. On the test set, GBM achieved the highest AUC of 0.779 (95% CI: 0.695–0.863), followed by NN (0.777) and LDA (0.771). Both GBM and LDA demonstrated acceptable calibration (Hosmer–Lemeshow *p* > 0.05) and clinical utility. SHAP analysis revealed that fasting insulin was the most influential indicator for LDA, while triglycerides dominated GBM case identification.

**Conclusion:**

This study developed interpretable machine learning models (LDA and GBM) for identifying MASLD in non-obese individuals using readily available clinical parameters. The models demonstrated acceptable discrimination and calibration, with SHAP analysis highlighting the central roles of insulin resistance and dyslipidemia in the pathogenesis of non-obese MASLD.

## Background

Metabolic dysfunction-associated steatotic liver disease (MASLD) is a chronic liver condition closely linked to metabolic dysregulation, with a global prevalence exceeding 30%, making it the most common cause of chronic liver disease worldwide (1, 2). While MASLD has been strongly associated with obesity, it is also highly prevalent in non-obese individuals with a normal body mass index (BMI), where its occurrence ranges from 15 to 20% (3, 4). Non-obese MASLD patients often exhibit a distinct phenotype, characterised by more pronounced insulin resistance, more severe dyslipidaemia, and a higher risk of progression to metabolic-dysfunction-associated steatohepatitis (MASH) and significant liver fibrosis (5, 6). Furthermore, MASLD significantly increases the risk of extrahepatic complications, including fatal and non-fatal cardiovascular events, chronic kidney disease, and type 2 diabetes, which represent leading causes of mortality in non-obese MASLD patients (7–9). Consequently, the development of early identification tools for MASLD specifically within non-obese populations has become an urgent and critical priority in hepatology research.

Liver biopsy, the histological gold standard for diagnosis, is unsuitable for large-scale screening due to its invasive nature, risk of sampling error, and high cost (10, 11). Abdominal ultrasonography, while widely used, has limited sensitivity for detecting mild steatosis, and its diagnostic accuracy is highly operator-dependent, leading to a substantial rate of missed diagnoses for early-stage disease (12, 13). More advanced non-invasive techniques, such as MRI-proton density fat fraction and vibration-controlled transient elastography with controlled attenuation parameter, offer higher accuracy but their widespread application in routine health assessments, particularly in resource-limited settings, is hindered by high costs and limited availability (14, 15). Even widely adopted non-invasive scores, such as the Fatty Liver Index (FLI), exhibit suboptimal diagnostic performance specifically in non-obese populations. A recent study demonstrated that the FLI had a remarkably low sensitivity of only 6.3% for detecting imaging-confirmed steatosis in lean individuals (16). Therefore, creating an objective, automated risk assessment tool using readily available clinical parameters from routine health check-ups holds significant potential for improving the early detection of MASLD in non-obese individuals.

Machine learning (ML) can efficiently process high-dimensional, multi-modal clinical data and capture complex non-linear relationships and interactions among variables, potentially revealing patterns beyond the scope of traditional statistical methods (17, 18). However, most existing MASLD diagnostic models have focused on obese or general populations (19, 20). Recent advances in explainable artificial intelligence, particularly the maturation of the SHapley Additive exPlanations (SHAP) framework, now enable the precise quantification of each variable’s contribution to a model’s identification (21). This provides an opportunity not only to build a diagnostic tool but also to gain insights into the pathogenic mechanisms underlying MASLD in non-obese individuals.

This study aimed to develop a non-invasive tool that leverages easily obtainable, multi-dimensional clinical parameters from standard health examinations to facilitate the rapid and accurate identification of MASLD in non-obese individuals. Such a tool could aid clinical decision-making for the prevention and management of MASLD in this specific population.

## Methods

### Participants

This study was reported in accordance with the Transparent Reporting of a Multivariable Prediction Model for Individual Prognosis or Diagnosis (TRIPOD) statement and the Strengthening the Reporting of Observational Studies in Epidemiology (STROBE) guidelines (22, 23) (Supplementary Files 1, 2). This study was performed with secondary data analysis (Ethical Approval No. 20250306-02). This project employed community-based recruitment to enroll participants, approved and implemented by the Medical Ethics Committee of the Sixth People’s Hospital of Qingdao. The original data collection period was from June to September 2025. All the original data collection processes strictly adhered to ethical norms and obtained the informed consent of the participants. The diagnosis of MASLD was established in accordance with the 2023 multi-society Delphi consensus, which requires the presence of hepatic steatosis in conjunction with at least one metabolic risk factor (24). Hepatic steatosis was defined as a controlled attenuation parameter (CAP) value ≥248 dB/m as per the EASL clinical practice guidelines. Exclusion criteria were: (1) body mass index (BMI) ≥ 25 kg/m^2^ (*n* = 1,088); (2) presence of other primary liver diseases, such as viral hepatitis, autoimmune liver disease (*n* = 46); (3) significant alcohol consumption: >140 g/week for females or >210 g/week for males (*n* = 201); (4) a confirmed history of liver diseases or previous liver surgery (*n* = 5); (5) patients whose data were incomplete or missing (*n* = 110); and (6) pregnancy (*n* = 5). After excluding 1,455 subjects based on the exclusion criteria, a final analysis included 768 subjects (Figure 1).

**Figure 1:**
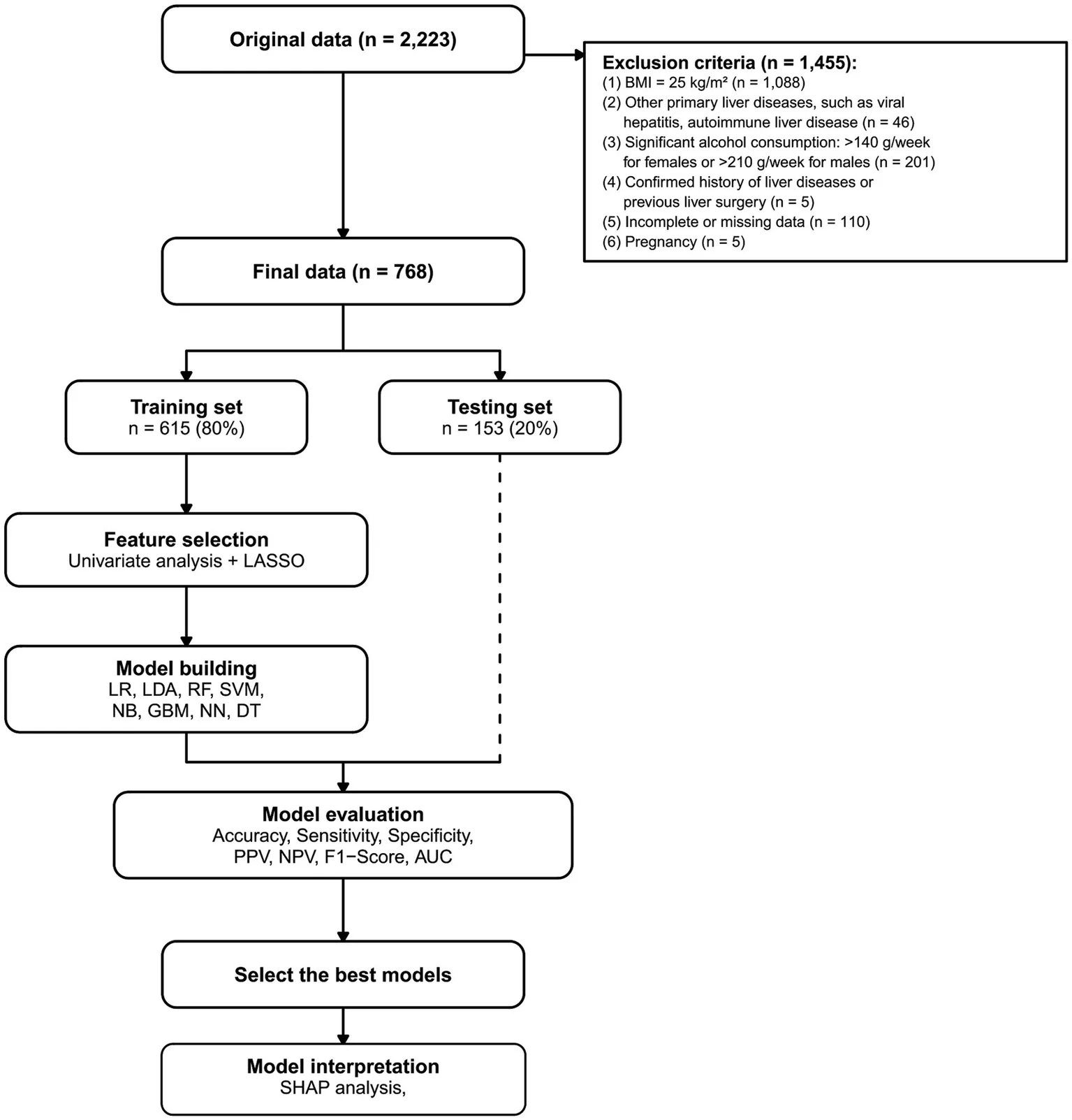
Flow chart of this study.

### Data collection

Demographic and clinical data were collected using standardized questionnaires, including age, sex, smoking status, alcohol consumption, and medical history (hypertension, diabetes mellitus and cardiovascular disease). Anthropometric measurements were performed by trained staff following standardized protocols. BMI was calculated as weight (kg) divided by height squared (m^2^). Laboratory tests were performed on peripheral venous blood samples collected after ≥8 h of overnight fasting. CAP was obtained using the FibroScan device. All the procedures were performed by experienced technicians following standard operating procedures.

### Statistical analysis and model development

All statistical analyses were performed using R software (version 4.5.2). *p* < 0.05 was considered statistically significant. Continuous variables are presented as mean ± SD and compared using independent *t*-test or Mann–Whitney *U* test as appropriate. Categorical variables are expressed as *n* (%) and compared using chi-square test. The dataset was randomly partitioned into a training set (80%, *n* = 615) and an independent test set (20%, *n* = 153) using stratified random sampling with a fixed seed (set.seed(42)) to ensure reproducibility. Feature selection was performed exclusively within the training set to prevent data leakage.

For feature selection, univariable analysis was first conducted to identify variables significantly associated with MASLD. Subsequently, Least Absolute Shrinkage and Selection Operator (LASSO) regression was employed to identify the most discriminative features and prevent overfitting. The optimal regularisation parameter (*λ*) was determined via 10-fold cross-validation. Two standard criteria for selecting *λ* were considered: *λ*.min, which minimises the mean cross-validated error, and *λ*.1se, which keeps the cross-validation error within one standard error. The *λ*.1se value was selected for the final model to enhance model sparsity and generalisability. The LASSO regression was implemented using the glmnet package in R (25).

Eight machine learning models were constructed based on selected indicators: Linear Discriminant Analysis (LDA), Gradient Boosting Machine (GBM), Neural Network (NN), Logistic Regression (LR), Support Vector Machine (SVM), Naïve Bayes (NB), Random Forest (RF), and Decision Tree (DT). All models were implemented and tuned using the caret package (version 6.0–94) in R. Model development and internal validation were performed on the training set using repeated stratified 10-fold cross-validation with 10 repetitions. For models requiring hyperparameter optimization, a limited automatic grid search was conducted using the default tuning grids provided by caret (tuneLength = 3). The complete specifications of software versions and the detailed hyperparameter grids for each model are comprehensively listed in Supplementary Table S1.

### Model evaluation and interpretability

The evaluation of model performance was based on a comprehensive set of metrics: the area under the receiver operating characteristic curve (AUC), accuracy, precision, recall (sensitivity), specificity, and the F1-score. Model calibration was evaluated using calibration slope, intercept, Brier score, and the Hosmer–Lemeshow goodness-of-fit test. Clinical utility was assessed using decision curve analysis (DCA) to evaluate net benefit across threshold probabilities. The discriminative performance of each model was compared against established clinical indices including the FLI, Hepatic Steatosis Index (HSI), and NAFLD Ridge Score (NRS) using the DeLong test for correlated ROC curves (26, 27). For interpretability analysis, the SHAP method was applied to the optimal model to quantify the magnitude and direction of each feature’s impact on identifications by calculating its marginal contribution, thereby ensuring transparency regarding the principal drivers of MASLD (28, 29).

## Results

### Baseline characteristics of the study population

A total of 768 non-obese participants were enrolled in this study. Among them, 226 individuals (29.4%) were diagnosed with MASLD. The cohort comprised 196 males (25.5%) and had a mean age of 50.6 ± 12.0 years. Comorbid conditions included hypertension (*n* = 73, 9.5%), diabetes mellitus (*n* = 65, 8.5%) and cardiovascular disease (*n* = 21, 2.7%). To develop and validate the machine learning models, the participants were randomly allocated into a training set (*n* = 615, 80%) and an independent testing set (*n* = 153, 20%) using stratified sampling. The baseline demographic, clinical, and laboratory characteristics were well-balanced between the training and testing sets, as evidenced by the absence of statistically significant differences in all variables (*p* > 0.05, Table 1).

**Table 1:** General characteristics of the study population.

Variable	Total (*n* = 768)	Training set (*n* = 615)	Testing set (*n* = 153)	*p* value
MASLD	226 (29.4%)	181 (29.4%)	45 (29.4%)	1.000
Sex (male)	196 (25.5%)	158 (25.7%)	38 (24.8%)	0.910
Age (years)	51 ± 12	50 ± 12	52 ± 12	0.174
Smoking history	77 (10.0%)	66 (10.7%)	11 (7.2%)	0.248
Hypertension	73 (9.5%)	60 (9.8%)	13 (8.5%)	0.748
Diabetes	65 (8.5%)	48 (7.8%)	17 (11.1%)	0.249
Cardiovascular disease	21 (2.7%)	17 (2.8%)	4 (2.6%)	1.000
Height (cm)	165.3 ± 7.7	165.4 ± 7.8	164.7 ± 7.5	0.275
Weight (kg)	59.7 ± 7.6	59.7 ± 7.7	59.5 ± 7.5	0.805
Systolic blood pressure (mmHg)	121 ± 17	121 ± 17	120 ± 15	0.211
Diastolic blood pressure (mmHg)	79 ± 10	80 ± 10	79 ± 10	0.715
Heart rate (bpm)	76 ± 11	76 ± 11	76 ± 10	0.483
Waist circumference (cm)	77.0 ± 8.3	76.9 ± 8.4	77.2 ± 7.7	0.660
Hip circumference (cm)	90.9 ± 6.2	90.9 ± 6.3	91.0 ± 5.8	0.827
Total bilirubin (μmol/L)	14.4 ± 6.7	14.3 ± 5.5	15.0 ± 10.2	0.362
Direct bilirubin (μmol/L)	4.7 ± 1.9	4.7 ± 2.0	4.8 ± 1.8	0.574
Indirect bilirubin (μmol/L)	9.70 ± 5.2	9.5 ± 3.7	10.2 ± 9.1	0.361
ALT (U/L)	19 ± 15	19 ± 16	19 ± 9	0.549
AST (U/L)	23 ± 10	23 ± 11	22 ± 6	0.190
ALT/AST ratio	0.82 ± 0.27	0.82 ± 0.28	0.84 ± 0.25	0.380
Albumin (g/L)	45.44 ± 2.11	45.42 ± 2.12	45.51 ± 2.09	0.618
LDL-C (mmol/L)	3.27 ± 0.92	3.28 ± 0.92	3.21 ± 0.93	0.422
Triglycerides (mmol/L)	1.25 ± 0.93	1.26 ± 0.97	1.20 ± 0.75	0.367
HDL-C (mmol/L)	1.54 ± 0.36	1.55 ± 0.36	1.52 ± 0.36	0.387
GGT (U/L)	30 ± 43	31 ± 47	25 ± 20	0.012
Total cholesterol (mmol/L)	5.38 ± 1.10	5.41 ± 1.10	5.28 ± 1.13	0.211
Lipoprotein(a) (mg/L)	194.11 ± 206.96	193.45 ± 207.42	196.75 ± 205.76	0.860
Apolipoprotein A1 (g/L)	1.32 ± 0.22	1.32 ± 0.22	1.30 ± 0.21	0.440
Apolipoprotein B (g/L)	0.88 ± 0.24	0.89 ± 0.24	0.87 ± 0.25	0.539
Alkaline phosphatase (U/L)	81 ± 25	82 ± 26	78 ± 21	0.102
Uric acid (μmol/L)	303 ± 76	304 ± 78	296 ± 66	0.203
Creatinine (μmol/L)	63 ± 16	63 ± 17	62 ± 14	0.768
Hemoglobin (g/L)	139 ± 15	139 ± 15	140 ± 14	0.182
WBC (10^9/L)	5.5 ± 1.5	5.5 ± 1.6	5.5 ± 1.4	0.525
RBC (10^12/L)	4.6 ± 0.4	4.6 ± 0.4	4.6 ± 0.4	0.612
Platelet (10^9/L)	247 ± 59	248 ± 59	245 ± 58	0.572
CRP (mg/L)	0.9 ± 1.6	0.9 ± 1.7	0.8 ± 1.2	0.128
β-2 microglobulin (mg/L)	1.48 ± 0.52	1.48 ± 0.53	1.47 ± 0.44	0.765
Cystatin C (mg/L)	0.98 ± 0.17	0.98 ± 0.17	0.98 ± 0.16	0.863
Fasting C-peptide (ng/mL)	1.60 ± 0.71	1.59 ± 0.74	1.61 ± 0.60	0.724
Fasting insulin (μIU/mL)	9.73 ± 4.72	9.60 ± 4.49	10.25 ± 5.55	0.176
Fasting plasma glucose (mmol/L)	5.30 ± 1.35	5.26 ± 1.19	5.48 ± 1.85	0.164
HbA1c (%)	5.72 ± 0.80	5.69 ± 0.73	5.82 ± 1.02	0.138

### Identification of key factors for MASLD

To identify the clinical indicators most strongly associated with MASLD and to mitigate the risk of overfitting, a two-step feature selection process was performed strictly within the training set. First, univariable logistic regression was applied to candidate clinical variables derived from the baseline measurements. This initial screening identified 27 variables that demonstrated a significant association with MASLD status (*p* < 0.05; complete results are available in Supplementary Table S2). Subsequently, to distill this set into a more concise and robust group of features, LASSO regression was employed. At the *λ*.1se value, LASSO regression ultimately retained eight variables with non-zero coefficients for subsequent model development. These selected features, along with their respective LASSO coefficients, were: apolipoprotein B, triglycerides, fasting insulin, fasting plasma glucose, waist circumference, age, alkaline phosphatase, and systolic blood pressure. The variable selection process via LASSO is visualized in Figure 2.

**Figure 2:**
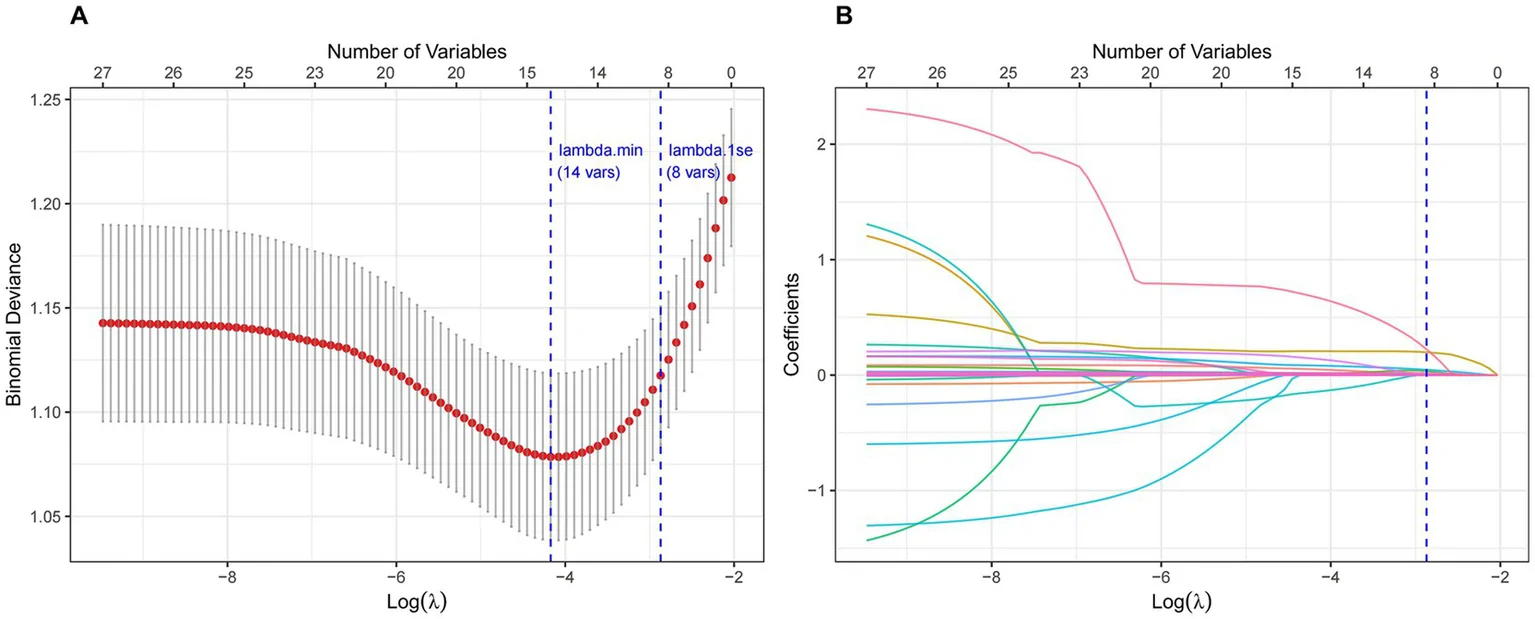
Feature selection using LASSO regression. **(A)** Cross-validation curves; **(B)** Lasso coefficient path plots for 27 variables.

### Comparison of machine learning model performance

Based on the eight key factors selected, eight distinct machine learning models were constructed. The AUC value for all models on the training and test sets are presented in Figures 3A,B, respectively. On the test set, GBM achieved the highest AUC (0.779, 0.695–0.863), followed by NN (0.777, 0.694–0.860) and LDA (0.771, 0.686–0.855). Complete performance metrics are presented in Table 2. In the testing set, calibration analysis showed that LDA (Hosmer-Lemeshow *p* = 0.609) and GBM (*p* = 0.502) exhibited acceptable calibration, whereas NB demonstrated significant miscalibration (*p* < 0.001) (Supplementary Table S3, Figure S1). DCA indicated that GBM and LDA provided superior net benefit across clinically relevant threshold probabilities (Supplementary Figure S2). DeLong test comparisons with established clinical scores (Supplementary Figure S3) revealed no statistically significant differences between machine learning models and FLI, HSI, or NRS (all *p* > 0.05), although GBM, NN, and LDA consistently achieved numerically higher AUCs than all three traditional indices. Based on comprehensive evaluation of discrimination, calibration, and clinical utility, GBM and LDA were identified as optimal models.

**Figure 3:**
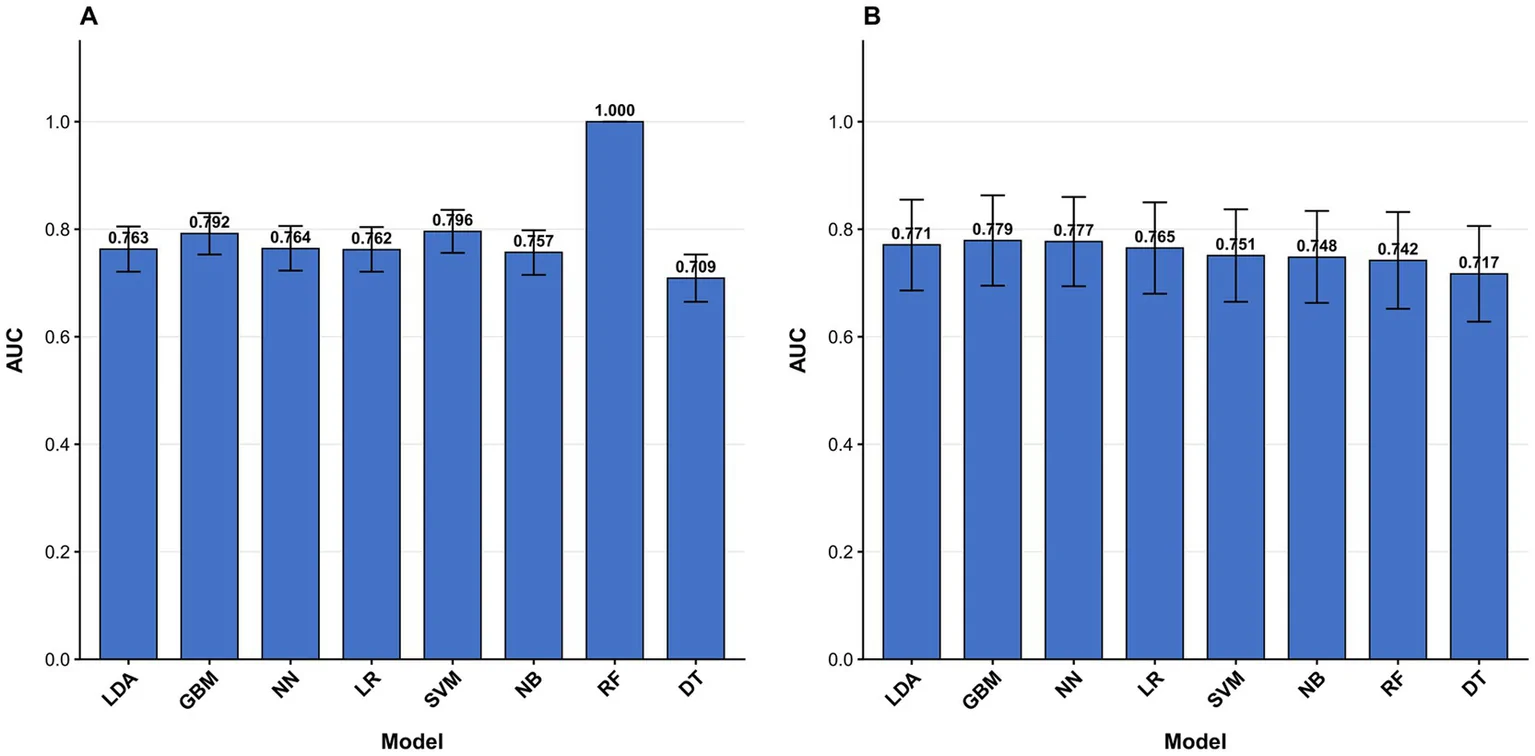
Comparison of AUC across machine learning models. **(A)** Training set (*n* = 615); **(B)** testing set (*n* = 153). LDA, Linear Discriminant Analysis; GBM, Gradient Boosting Machine; NN, Neural Network; LR, Logistic Regression; SVM, Support Vector Machine; NB, Naïve Bayes; RF, Random Forest; DT, Decision Tree.

**Table 2:** Diagnostic performance of the eight models for MASLD.

Model	AUC (95% CI)	Sensitivity (95% CI)	Specificity (95% CI)	F1-score (95% CI)	Accuracy (95% CI)	PPV (95% CI)	NPV (95% CI)	PLR (95% CI)	NLR (95% CI)
Training set									
LDA	0.763 (0.721–0.805)	0.343 (0.277–0.414)	0.924 (0.895–0.945)	0.449 (0.379–0.521)	0.753 (0.717–0.785)	0.653 (0.553–0.741)	0.771 (0.733–0.805)	4.50 (3.07–6.62)	0.71 (0.64–0.79)
GBM	0.792 (0.753–0.830)	0.398 (0.329–0.471)	0.935 (0.908–0.955)	0.512 (0.444–0.578)	0.777 (0.743–0.808)	0.720 (0.625–0.799)	0.788 (0.751–0.821)	6.17 (4.13–9.20)	0.64 (0.57–0.73)
NN	0.764 (0.723–0.806)	0.431 (0.361–0.504)	0.873 (0.839–0.901)	0.497 (0.434–0.561)	0.743 (0.707–0.776)	0.586 (0.501–0.667)	0.786 (0.748–0.821)	3.40 (2.52–4.58)	0.65 (0.57–0.74)
LR	0.762 (0.721–0.804)	0.331 (0.267–0.403)	0.919 (0.890–0.941)	0.435 (0.365–0.505)	0.746 (0.710–0.779)	0.632 (0.531–0.722)	0.767 (0.729–0.802)	4.11 (2.81–6.01)	0.73 (0.65–0.81)
SVM	0.796 (0.756–0.836)	0.376 (0.308–0.448)	0.947 (0.922–0.964)	0.500 (0.426–0.569)	0.779 (0.744–0.810)	0.747 (0.649–0.825)	0.784 (0.747–0.817)	7.09 (4.57–11.01)	0.66 (0.59–0.74)
NB	0.757 (0.715–0.798)	0.370 (0.303–0.443)	0.903 (0.872–0.928)	0.462 (0.391–0.531)	0.746 (0.710–0.779)	0.615 (0.521–0.701)	0.775 (0.736–0.809)	3.83 (2.71–5.40)	0.70 (0.62–0.78)
RF	1.000 (1.000–1.000)	1.000 (0.979–1.000)	1.000 (0.991–1.000)	1.000 (1.000–1.000)	1.000 (0.994–1.000)	1.000 (0.979–1.000)	1.000 (0.991–1.000)	Inf (NA–NA)	0.00 (NA–NA)
DT	0.709 (0.665–0.753)	0.387 (0.319–0.459)	0.924 (0.895–0.945)	0.493 (0.423–0.563)	0.766 (0.731–0.798)	0.680 (0.584–0.762)	0.783 (0.745–0.817)	5.09 (3.49–7.41)	0.66 (0.59–0.75)
Testing set									
LDA	0.771 (0.686–0.855)	0.333 (0.214–0.479)	0.944 (0.884–0.974)	0.455 (0.290–0.592)	0.765 (0.692–0.825)	0.714 (0.500–0.862)	0.773 (0.694–0.836)	6.00 (2.49–14.47)	0.71 (0.57–0.87)
GBM	0.779 (0.695–0.863)	0.400 (0.270–0.545)	0.917 (0.849–0.956)	0.500 (0.344–0.633)	0.765 (0.692–0.825)	0.667 (0.478–0.814)	0.786 (0.706–0.848)	4.80 (2.33–9.87)	0.65 (0.51–0.84)
NN	0.777 (0.694–0.860)	0.333 (0.214–0.479)	0.935 (0.872–0.968)	0.448 (0.295–0.592)	0.758 (0.685–0.819)	0.682 (0.473–0.836)	0.771 (0.692–0.835)	5.14 (2.25–11.76)	0.71 (0.58–0.88)
LR	0.765 (0.680–0.850)	0.289 (0.177–0.434)	0.954 (0.896–0.980)	0.413 (0.241–0.553)	0.758 (0.685–0.819)	0.722 (0.491–0.875)	0.763 (0.685–0.827)	6.24 (2.36–16.48)	0.75 (0.62–0.90)
SVM	0.751 (0.665–0.837)	0.267 (0.160–0.410)	0.954 (0.896–0.980)	0.387 (0.222–0.535)	0.752 (0.678–0.813)	0.706 (0.469–0.867)	0.757 (0.679–0.822)	5.76 (2.15–15.40)	0.77 (0.64–0.92)
NB	0.748 (0.663–0.834)	0.400 (0.270–0.545)	0.907 (0.838–0.949)	0.493 (0.338–0.625)	0.758 (0.685–0.819)	0.643 (0.458–0.793)	0.784 (0.704–0.847)	4.32 (2.17–8.62)	0.66 (0.52–0.85)
RF	0.742 (0.652–0.832)	0.333 (0.214–0.479)	0.926 (0.861–0.962)	0.441 (0.277–0.587)	0.752 (0.678–0.813)	0.652 (0.449–0.812)	0.769 (0.690–0.833)	4.50 (2.05–9.86)	0.72 (0.58–0.89)
DT	0.717 (0.628–0.806)	0.444 (0.309–0.588)	0.935 (0.872–0.968)	0.556 (0.394–0.686)	0.791 (0.720–0.848)	0.741 (0.553–0.868)	0.802 (0.723–0.862)	6.86 (3.12–15.07)	0.59 (0.46–0.78)

LDA, Linear Discriminant Analysis; GBM, Gradient Boosting Machine; NN, Neural Network; LR, Logistic Regression; SVM, Support Vector Machine; NB, Naïve Bayes; RF, Random Forest; DT, Decision Tree.

### Model interpretability analysis

SHAP analysis was performed on GBM and LDA in testing set (Figure 4, Supplementary Table S4). For LDA, fasting insulin demonstrated the highest mean absolute SHA*p* value, followed by waist circumference, apolipoprotein B, and triglycerides. For GBM, triglycerides exhibited the highest contribution, followed by fasting insulin, waist circumference, and age. The beeswarm plots illustrated that higher values of metabolic associated factors consistently shifted model identifications towards the “MASLD-positive” class.

**Figure 4:**
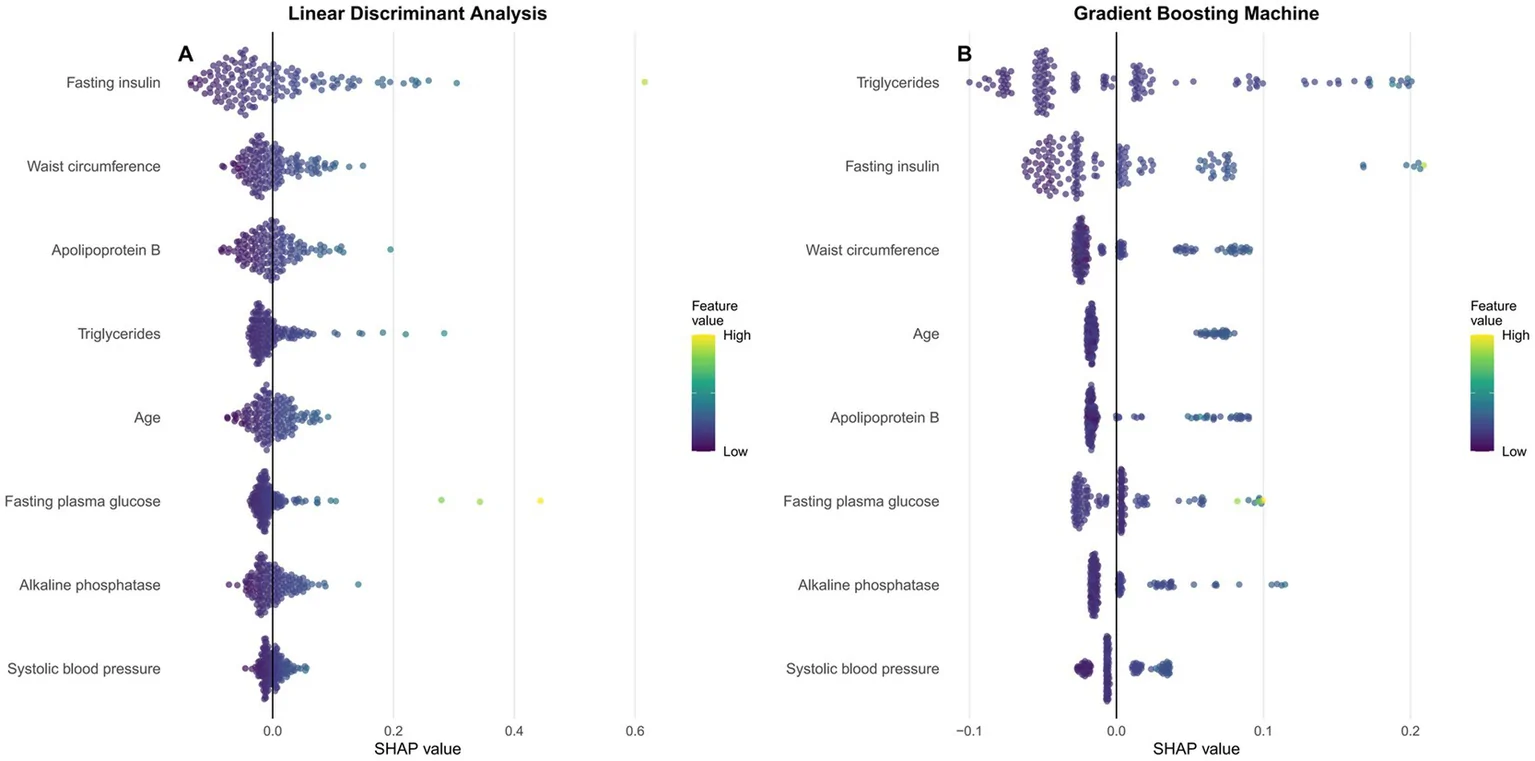
SHAP beeswarm plots for LDA **(A)** and GBM **(B)**. Each point represents an individual patient; color indicates feature value. Features are ranked by mean absolute SHAP value.

## Discussion

This study developed and validated interpretable machine learning models for identifying MASLD in a non-obese population. Among eight algorithms evaluated, GBM and LDA showed a balanced profile in terms of discriminative ability (AUC = 0.779 and 0.771), calibration quality, and clinical utility within our cohort. To our knowledge, this is among the first studies to systematically apply multiple machine learning algorithms combined with SHAP-based interpretability analysis for MASLD identification specifically in non-obese individuals.

A central finding of this study is the dominant role of insulin resistance and dyslipidaemia in the identification of MASLD among non-obese individuals. SHAP analysis revealed that fasting insulin was the most influential factor in the LDA model, while triglycerides dominated GBM identifications. Even in individuals with normal BMI, insulin resistance remains a critical driver of hepatic lipid accumulation and progression to steatohepatitis (30, 31). Previous studies have reported that non-obese MASLD patients commonly exhibit more severe dysregulation of both glucose and lipid metabolism compared to their healthy counterparts (3, 32). Elevated TG levels reflect increased hepatic *de novo* lipogenesis and/or impaired lipid clearance, directly promoting hepatic steatosis (33). The concordance of our data-driven SHAP results with known pathobiological mechanisms strengthens the biological plausibility and clinical credibility of the proposed models.

Waist circumference emerged as an important factor in both GBM and LDA, consistent with accumulating evidence that visceral adiposity, rather than overall obesity, is the primary driver of metabolic complications in non-obese individuals (4, 34). This finding has direct implications for clinical practice, suggesting that waist circumference measurement should be routinely incorporated into the identification of MASLD even in normal-weight populations.

This study confirms the important value of blood pressure and liver enzyme markers in risk stratification. Both SBP and ALP were selected as key factors. Elevated SBP is often associated with sympathetic nervous system activation and renin-angiotensin system dysregulation, which are also known to promote hepatic stellate cell activation and liver inflammation (35, 36). Furthermore, a study by Yuan et al. (37), which integrated observational data from the NHANES 2017–2018 cohort with a two-sample Mendelian randomization approach, demonstrated a causal association between hypertension and an increased risk of non-alcoholic fatty liver disease. An elevated ALP level may suggest underlying cholestasis or a ductular reaction, which is associated with specific pathological subtypes of MASLD, such as steatohepatitis with ductular reaction, thereby enriching the pathobiological interpretation of our model (38). Additionally, ALP has been incorporated as a diagnostic factor for MASLD incidence and prognosis in several previous studies (39, 40).

Regarding model selection, our results demonstrate that parsimonious models can achieve competitive or superior performance compared to more complex algorithms in moderate-sized clinical datasets. LDA, a linear classifier with minimal hyperparameters, achieved an AUC of 0.771 on the independent test set, comparable to GBM (0.779) and NN (0.777), consistent with the bias-variance trade-off principle whereby simpler models benefit from lower variance in settings with limited sample size (41). RF achieved perfect training discrimination (AUC = 1.000) but degraded test performance (AUC = 0.742), exemplifying overfitting in bagging-based ensembles (42). In contrast, GBM’s sequential boosting with shrinkage effectively controlled complexity while maintaining acceptable calibration (Hosmer-Lemeshow *p* = 0.502). DeLong test comparisons with the established clinical indices, including the FLI, HSI and NRS, showed that GBM, NN, and LDA consistently achieved numerically higher AUCs than all three indices, though the differences did not reach conventional statistical significance (all *p* > 0.05), likely attributable to the limited test set size (*n* = 153, 45 cases). Nevertheless, AUC improvements of 2.4–5.6% points represent clinically meaningful gains in community screening settings where even modest improvements can substantially reduce misclassification at population scale. Larger cohorts are expected to increase statistical power and may render these differences significant.

A methodological strength of this study is the systematic construction and comparison of multiple machine learning algorithms, combined with the deep integration of explainable artificial intelligence techniques. The application of SHAP analysis to interpret the contribution of individual features to the MASLD identifications significantly increases the model’s trustworthiness and potential for clinical adoption. However, several limitations must be acknowledged. First, this was a single-centre, cross-sectional study; external validation in geographically and ethnically diverse multicentre cohorts is essential to confirm generalizability. Second, the diagnosis of MASLD was based on abdominal ultrasonography, a non-invasive modality with lower sensitivity for mild steatosis compared to the histological gold standard of liver biopsy, which may lead to disease misclassification. Third, the cohort included patients with diagnosed and treated conditions such as type 2 diabetes and hypertension. Pharmacological management (e.g., with hypoglycemic agents, statins, or antihypertensives) may have partially normalized key metabolic parameters in these individuals, potentially attenuating their discriminative power in the model and representing a source of unmeasured confounding. Fourth, although we rigorously excluded other known liver diseases, the potential for residual confounding factors cannot be entirely ruled out. Fifth, the optimal models showed low sensitivity (33–40%) on the test set, meaning many MASLD cases would be missed at the default threshold. Future studies should consider threshold optimization or resampling strategies to improve detection for screening purposes. Finally, potentially important determinants of MASLD, such as detailed dietary patterns, physical activity levels, and genetic markers, were not included. Future research integrating multi-omics data and detailed medication history holds promise for further refining the model’s accuracy and biological insight.

## Conclusion

This study developed interpretable machine learning models (LDA and GBM) for identifying MASLD in non-obese individuals using eight routine clinical parameters. Both models demonstrated acceptable discrimination, good calibration, and clinical utility. SHAP analysis revealed that fasting insulin and triglycerides were the most influential features, highlighting the central pathogenic roles of insulin resistance and dyslipidaemia in non-obese MASLD.

## Data Availability

The data analyzed in this study is subject to the following licenses/restrictions: requests to access the datasets should be directed to the corresponding authors (qphccnk@163.com).
