# Supplementary material for: The application of explainable machine learning for the identification of MASLD in non-obese populations
Source: Front Public Health. 2026 Sep 28;14:1851486. doi: 10.3389/fpubh.2026.1851486 (PMC13655027; doi:10.3389/fpubh.2026.1851486)
Supplement: Supplementary file 3 [file Supplementary_file_3.DOCX]

Table S1 Detailed implementation and hyperparameter specifications for the machine learning models

| **Model** | **Parameter** | **Value/Search Grid** |
| --- | --- | --- |
| LDA | Implementation | MASS::lda |
|  | Method | moment |
| GBM | Implementation | gbm via caret |
|  | n.trees | 50, 100, 150 |
|  | interaction.depth | 1, 2, 3 |
|  | shrinkage | 0.1 |
|  | n.minobsinnode | 10 |
|  | bag.fraction | 0.5 |
| NN | Implementation | nnet |
|  | Hidden Layers | 1 |
|  | Hidden Units (size) | 1, 3, 5 |
|  | Activation Function | Sigmoid (logistic) |
|  | Weight Decay | 0, 0.0001, 0.001, 0.01, 0.1 |
|  | Max Iterations | 100 |
| LR | Implementation | stats::glm |
|  | Family | binomial |
| SVM | Implementation | kernlab::ksvm |
|  | Kernel | Radial Basis Function |
|  | sigma | Estimated via sigest() |
|  | Cost (C) | 0.25, 0.5, 1, 2 |
| NB | Implementation | naivebayes::naive_bayes |
|  | usekernel | TRUE/FALSE |
|  | laplace | 0, 1 |
| RF | Implementation | randomForest |
|  | ntree | 500 |
|  | mtry | 2, 4, 8 |
|  | nodesize | 1 |
| DT | Implementation | rpart |
|  | Complexity Parameter (cp) | Automatic 3-value grid |
|  | Split Criterion | Gini Index |
|  | minsplit | 20 |

Note on Hyperparameter Tuning: For all models requiring optimization, an automatic grid search was conducted using the default parameter grids provided by the caret package, with the number of candidate values per parameter set by tuneLength = 3. No manual or advanced (e.g., Bayesian) optimization was performed. LDA: Linear Discriminant Analysis; GBM: Gradient Boosting Machine; NN: Neural Network; LR: Logistic Regression; SVM: Support Vector Machine; NB: Naïve Bayes; RF: Random Forest; DT: Decision Tree.

Table S2 Univariate analysis of MASLD in the training set

| **Variable** | **MAFLD (n=181)** | **Non-MASLD (n=434)** | **P value** |
| --- | --- | --- | --- |
| Sex (Male) | 51 (28.2) | 107 (24.7) | 0.364 |
| Age (years) | 54 ± 12 | 49 ± 12 | <0.001 |
| Smoking history | 30 (16.6) | 36 (8.3) | 0.004 |
| Tumor history | 1 (0.6) | 0 (0.0) | 0.294 |
| Hypertension | 23 (12.7) | 37 (8.5) | 0.135 |
| Diabetes | 24 (13.3) | 24 (5.5) | 0.003 |
| Cardiovascular disease | 4 (2.2) | 13 (3.0) | 0.789 |
| Height (cm) | 166.0 ± 8.7 | 165.2 ± 7.4 | 0.660 |
| Weight (kg) | 62.0 ± 7.6 | 58.7 ± 7.5 | <0.001 |
| Systolic BP (mmHg) | 127 ± 18 | 119 ± 17 | <0.001 |
| Diastolic BP (mmHg) | 82 ± 10 | 79 ± 10 | <0.001 |
| Heart rate (bpm) | 77 ± 11 | 76 ± 11 | 0.080 |
| Waist circumference (cm) | 80.2 ± 8.0 | 75.6 ± 8.2 | <0.001 |
| Hip circumference (cm) | 92.1 ± 6.0 | 90.4 ± 6.3 | 0.003 |
| Total bilirubin (umol/L) | 14.2 ± 5.4 | 14.3 ± 5.5 | 0.997 |
| Direct bilirubin (umol/L) | 4.6 ± 1.8 | 4.8 ± 2.0 | 0.329 |
| Indirect bilirubin (umol/L) | 9.6 ± 3.8 | 9.5 ± 3.7 | 0.631 |
| ALT (U/L) | 22 ± 20 | 18 ± 14 | 0.001 |
| AST (U/L) | 24 ± 14 | 22 ± 10 | 0.369 |
| ALT/AST ratio | 0.89 ± 0.33 | 0.79 ± 0.25 | <0.001 |
| Albumin (g/L) | 45.42 ± 2.09 | 45.42 ± 2.13 | 0.695 |
| LDL-C (mmol/L) | 3.52 ± 0.92 | 3.18 ± 0.90 | <0.001 |
| Triglycerides (mmol/L) | 1.70 ± 1.33 | 1.08 ± 0.71 | <0.001 |
| HDL-C (mmol/L) | 1.43 ± 0.33 | 1.60 ± 0.36 | <0.001 |
| GGT (U/L) | 46 ± 74 | 25 ± 27 | <0.001 |
| Total cholesterol (mmol/L) | 5.61 ± 1.09 | 5.32 ± 1.09 | <0.001 |
| Lipoprotein(a) (mg/L) | 181.13 ± 201.82 | 198.59 ± 209.73 | 0.104 |
| Apolipoprotein A1 (g/L) | 1.30 ± 0.22 | 1.33 ± 0.22 | 0.134 |
| Apolipoprotein B (g/L) | 0.97 ± 0.26 | 0.85 ± 0.23 | <0.001 |
| ALP (U/L) | 89 ± 26 | 78 ± 25 | <0.001 |
| Uric acid (umol/L) | 329 ± 90 | 294 ± 70 | <0.001 |
| Creatinine (umol/L) | 62 ± 13 | 63 ± 18 | 0.937 |
| Hemoglobin (g/L) | 142 ± 15 | 137 ± 15 | <0.001 |
| WBC (10^9/L) | 5.7 ± 1.5 | 5.3 ± 1.5 | <0.001 |
| RBC (10^12/L) | 4.7 ± 0.4 | 4.6 ± 0.4 | 0.069 |
| Platelet (10^9/L) | 254 ± 53 | 245 ± 61 | 0.057 |
| CRP (mg/L) | 1.3 ± 2.3 | 0.8 ± 1.4 | <0.001 |
| Beta-2 microglobulin (mg/L) | 1.60 ± 0.71 | 1.44 ± 0.43 | <0.001 |
| Cystatin C (mg/L) | 1.01 ± 0.16 | 0.96 ± 0.17 | 0.001 |
| Fasting C-peptide (ng/mL) | 1.82 ± 0.71 | 1.49 ± 0.73 | <0.001 |
| Fasting insulin (uIU/mL) | 11.45 ± 5.43 | 8.82 ± 3.78 | <0.001 |
| Glucose (mmol/L) | 5.65 ± 1.73 | 5.10 ± 0.82 | <0.001 |
| HbA1c (%) | 5.90 ± 1.02 | 5.60 ± 0.55 | <0.001 |

Table S3 Calibration Performance Metrics of Eight Machine Learning Models in the testing set

| Model | Slope | Intercept | Brier | H-L χ² | H-L P-value |
| --- | --- | --- | --- | --- | --- |
| LDA | 1.101 | 0.084 | 0.164 | 6.34 | 0.609 |
| GBM | 1.294 | 0.245 | 0.164 | 7.33 | 0.502 |
| NN | 1.189 | 0.179 | 0.165 | 7.84 | 0.449 |
| LR | 1.150 | 0.088 | 0.166 | 5.46 | 0.708 |
| SVM | 1.235 | 0.204 | 0.177 | 5.99 | 0.648 |
| NB | 0.275 | -0.469 | 0.199 | 246.77 | <0.001 |
| RF | 0.958 | -0.070 | 0.174 | 4.50 | 0.809 |
| DT | 1.100 | 0.066 | 0.166 | Not applicable | Not applicable |

H-L: The Hosmer-Lemeshow test; LDA: Linear Discriminant Analysis; GBM: Gradient Boosting Machine; NN: Neural Network; LR: Logistic Regression; SVM: Support Vector Machine; NB: Naïve Bayes; RF: Random Forest; DT: Decision Tree.

Table S4 The mean absolute SHAP value of the 8 variables in LDA and GBM.

| Rank | LDA Feature | LDA mean absolute SHAP value | GBM Feature | GBM mean absolute SHAP value |
| --- | --- | --- | --- | --- |
| 1 | Fasting insulin | 0.07484 | Triglycerides | 0.06246 |
| 2 | Waist circumference | 0.03459 | Fasting insulin | 0.04371 |
| 3 | Apolipoprotein B | 0.03222 | Waist circumference | 0.03009 |
| 4 | Triglycerides | 0.02822 | Age | 0.02752 |
| 5 | Age | 0.02668 | Apolipoprotein B | 0.02648 |
| 6 | Glucose | 0.02600 | Glucose | 0.02120 |
| 7 | Alkaline phosphatase | 0.02422 | Alkaline phosphatase | 0.01919 |
| 8 | Systolic blood pressure | 0.01406 | Systolic blood pressure | 0.01457 |

SHAP: SHapley Additive exPlanations; LDA: Linear Discriminant Analysis; GBM: Gradient Boosting Machine.

Figure S1 Calibration curves of eight machine learning models in Testing set. A: LDA; B: GBM; C: NN; D: LR; E: SVM; F: NB; G: RF; H: DT. LDA: Linear Discriminant Analysis; GBM: Gradient Boosting Machine; NN: Neural Network; LR: Logistic Regression; SVM: Support Vector Machine; NB: Naïve Bayes; RF: Random Forest; DT: Decision Tree.

Figure S2 Decision Curve Analysis of eight machine learning models in the testing set. LDA: Linear Discriminant Analysis; GBM: Gradient Boosting Machine; NN: Neural Network; LR: Logistic Regression; SVM: Support Vector Machine; NB: Naïve Bayes; RF: Random Forest; DT: Decision Tree.

Figure S3 Pairwise DeLong test for differences in AUC among machine learning models, FLI, HSI, and NRS in the testing set. LDA: Linear Discriminant Analysis; GBM: Gradient Boosting Machine; NN: Neural Network; LR: Logistic Regression; SVM: Support Vector Machine; NB: Naïve Bayes; RF: Random Forest; DT: Decision Tree; FLI: Fatty Liver Index; HSI: Hepatic Steatosis Index; NRS: NAFLD Ridge Score.
